# Myeloid-derived Wnts play an indispensible role in macrophage and fibroblast activation and kidney fibrosis

**DOI:** 10.7150/ijbs.94166

**Published:** 2024-04-08

**Authors:** Yuan Tian, Jiongcheng Chen, Wenshu Huang, Qian Ren, Junxia Feng, Jinlin Liao, Haiyan Fu, Lili Zhou, Youhua Liu

**Affiliations:** 1 State Key Laboratory of Organ Failure Research, Division of Nephrology, Nanfang Hospital, Southern Medical University, Guangzhou, China.; 2 Department of Nephrology, Jingzhou Hospital Affiliated to Yangze University, Jingzhou, China.; 3 National Clinical Research Center of Kidney Disease, Guangdong Provincial Institute of Nephrology, Guangzhou, China.

**Keywords:** Macrophage, Wnt, Wntless, fibroblast activation, kidney fibrosis

## Abstract

Wnt/β-catenin signaling plays a pivotal role in the pathogenesis of chronic kidney diseases (CKD), which is associated with macrophage activation and polarization. However, the relative contribution of macrophage-derived Wnts in the evolution of CKD is poorly understood. Here we demonstrate a critical role of Wnts secreted by macrophages in regulating renal inflammation and fibrosis after various injuries. In mouse model of kidney fibrosis induced by unilateral ureteral obstruction (UUO), macrophages were activated and polarized to M1 and M2 subtypes, which coincided with the activation of Wnt/β-catenin signaling. *In vitro*, multiple Wnts were induced in primary cultured bone marrow-derived macrophages (BMDMs) after polarization. Conversely, Wnt proteins also stimulated the activation and polarization of BMDMs to M1 and M2 subtype. Blockade of Wnt secretion from macrophages in mice with myeloid-specific ablation of Wntless (Wls), a cargo receptor that is obligatory for Wnt trafficking and secretion, blunted macrophage infiltration and activation and inhibited the expression of inflammatory cytokines. Inhibition of Wnt secretion by macrophages also abolished β-catenin activation in tubular epithelium, repressed myofibroblast activation and reduced kidney fibrosis after either obstructive or ischemic injury. Furthermore, conditioned medium from Wls-deficient BMDMs exhibited less potency to stimulate fibroblast proliferation and activation, compared to the controls. These results underscore an indispensable role of macrophage-derived Wnts in promoting renal inflammation, fibroblasts activation and kidney fibrosis.

## Introduction

Kidney fibrosis is the common pathological feature of a wide variety of chronic kidney diseases (CKD), which is characterized by renal inflammation, tubular atrophy and fibroblast activation 1-3. The activation and expansion of interstitial fibroblasts eventually cause excessive production of the extracellular matrix (ECM) components such as collagens and fibronectin, leading to tissue scarring and destruction of normal kidney structure 4, 5. Kidney fibrosis is generally considered as an irreversible process, and there is no specific therapy that can completely halts its progression 6-8. As such, it is paramount to better understand the underlying mechanism of renal fibrosis for developing effective treatment strategies. Extensive studies demonstrate that macrophage activation and polarization are closely associated with an increased expansion of interstitial fibroblasts, progressive tissue fibrosis and poor outcome of CKD 9, 10. However, exactly how macrophage polarization is connected to fibroblast activation and tissue fibrosis in CKD remains largely unknown.

Macrophages are a heterogenous population of tissue-resident cells. They often possess phagocytic ability by cleaning dead cells and debris and produce various cytokines, chemokines and other soluble factors 11-13. They are originated from progenitor mononuclear phagocyte system within bone marrow and migrated into tissues. Upon activation, the tissue-resident macrophages are polarized to classically activated M1 subtype macrophages, which could switch to alternatively activated M2 subtype macrophages along with disease progression. Both subtypes of macrophages could secret pathogenic chemokines and cytokines, such as interleukin-1 (IL-1), IL-6 and tumor necrosis factor-α (TNF-α) in the M1 and IL-10, transforming growth factor-β (TGF-β1) and fibroblast growth factor-2 (FGF-2) in the M2, which mediate proinflammatory and profibrotic effects in kidney diseases 14-16. Earlier studies have shown that Wnt7b is produced by macrophages to stimulate tubular repair and regeneration after acute kidney injury (AKI) 17. However, whether Wnt ligands produced by macrophages play a role in the pathogenesis of CKD is poorly understood.

Wnt ligands comprise a large family of secreted, hydrophobic glycoproteins 18, 19. In various forms of CKD, many Wnt ligands are induced simultaneously in different types of kidney cells such as tubular epithelial cells, interstitial fibroblasts and glomerular podocytes 20, 21. As secretory proteins, the intracellular trafficking and secretion of Wnt ligands in the producing cells are tightly controlled by Wntless (Wls), also known as Evenness Interrupted (Evi) in *Drosophila*. Wls is an evolutionarily conserved seven-pass transmembrane protein, which functions as a cargo receptor and is obligatory for the secretion of Wnt proteins 22, 23. Because Wls controls the release of all Wnts from the producing cells, conditional deletion of Wls would enable cell type-specific control of Wnt secretion *in vivo*. Knockout of Wls in specific cell types such as tubular epithelial cells, interstitial fibroblasts and glomerular podocytes in the kidney has shown that the lack of Wls blocks Wnt secretion from these cells 24, 25. However, the impact of blocking Wnt secretion from macrophages in the kidney has not been reported.

In this study, we examined the role of myeloid-derived Wnts in regulating macrophage polarization, fibroblast activation and kidney fibrosis *in vivo* and *in vitro*. We showed that both M1 and M2 polarized macrophages produce an increased amount of Wnt ligands, which in turn induce further activation of M1 and M2 macrophages. Our studies indicate that myeloid-derived Wnts play a pivotal role in mediating macrophage activation and kidney fibrosis.

## Results

### Macrophage activation is associated with Wnt induction in fibrotic kidney

**Figure 1:**
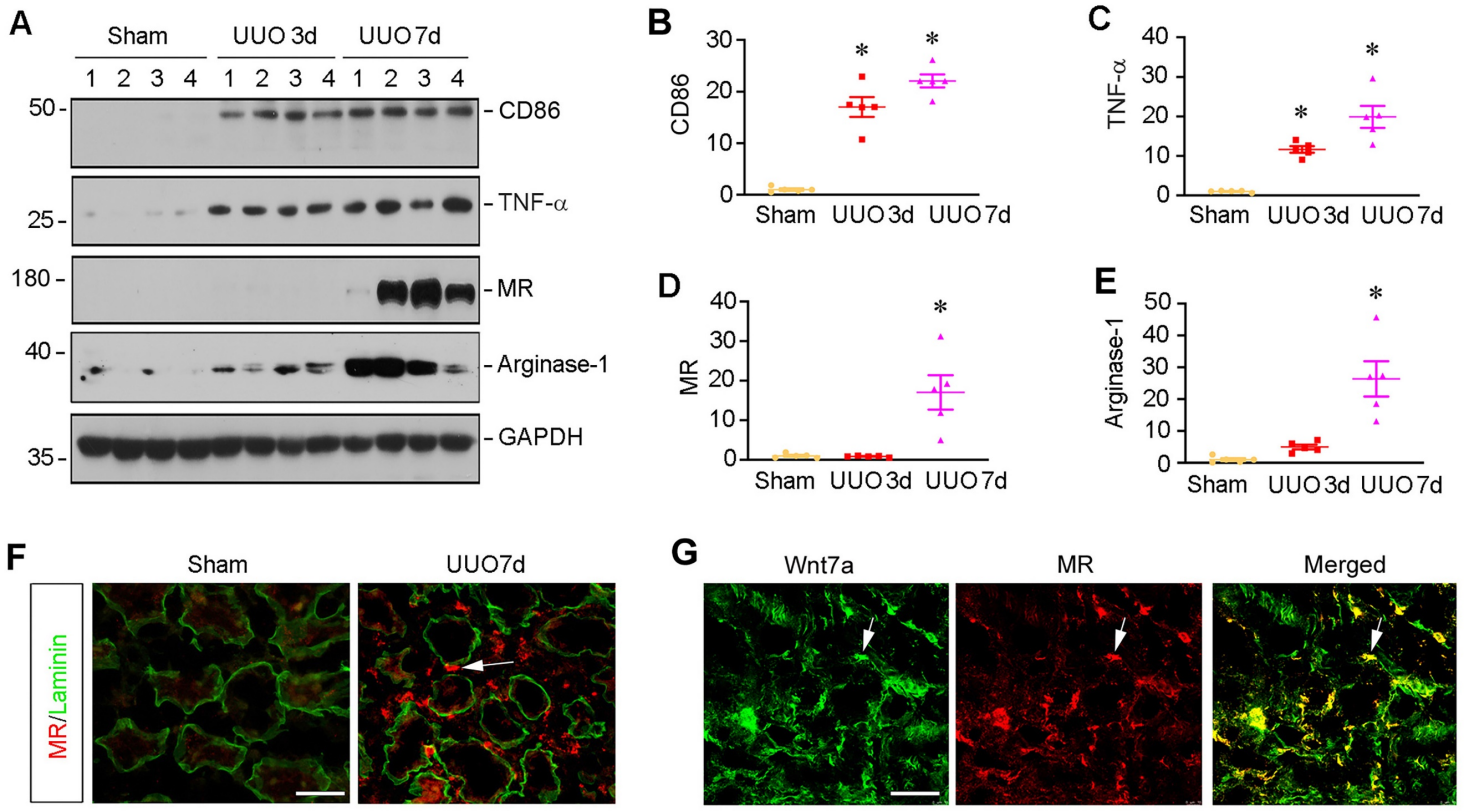
**Macrophage activation in chronic kidney disease is associated with Wnt induction.** (**A**) Western blot analyses show the expression of macrophage-related markers such as CD86, TNF-α, mannose receptor (MR) and arginase-1 in the kidney at 0, 3, and 7 days after UUO. (**B**-**E**) Quantitative data of the relative abundance of various proteins at different times as indicated. *P < 0.05 versus sham group (n=5). (**F**) Immunofluorescence staining demonstrated MR (red) expression in sham and UUO (7 day) of kidney sections. The sections were also stained for laminin (green) to outline tubular compartment. (**G**) Representative immunofluorescence staining showed that Wnt7a was induced in those MR-positive cells. Frozen sections of UUO kidney were costained with antibodies against Wnt7a (green) and MR (red). Arrows indicate positive staining. Scale bar, 50 µm.

To investigate macrophage activation in CKD, we examined the molecular signatures of macrophage polarization in mouse model of kidney fibrosis induced by unilateral ureteral obstruction (UUO). As shown in Figure 1A-C, markers for M1 subtype macrophage polarization such as CD86, also known as B7-2, and TNF-α were quickly induced, starting from 3 days after UUO, as shown by Western blot analysis of whole kidney lysates. We then analyzed the expressions of mannose receptor (MR) and arginase-1, two principal M2 macrophage polarization markers. Compared to the induction of M1 macrophage, the markers of M2 macrophages such as MR and aginase-1 were mainly upregulated at 7 days after UUO (Figure 1A, D and E). Consistently, co-immunofluorescence staining for MR and laminin showed that MR-positive macrophages were upregulated in renal interstitial compartment at 7 days after UUO (Figure 1F). Interestingly, we observed the colocalization of MR and Wnt7a in the obstructed kidney at 7 days after UUO (Figure 1G), suggesting that macrophage activation and polarization in CKD is associated with the activation of Wnt/β-catenin signaling.

### Wnts mediate macrophage activation and polarization *in vitro*

**Figure 2:**
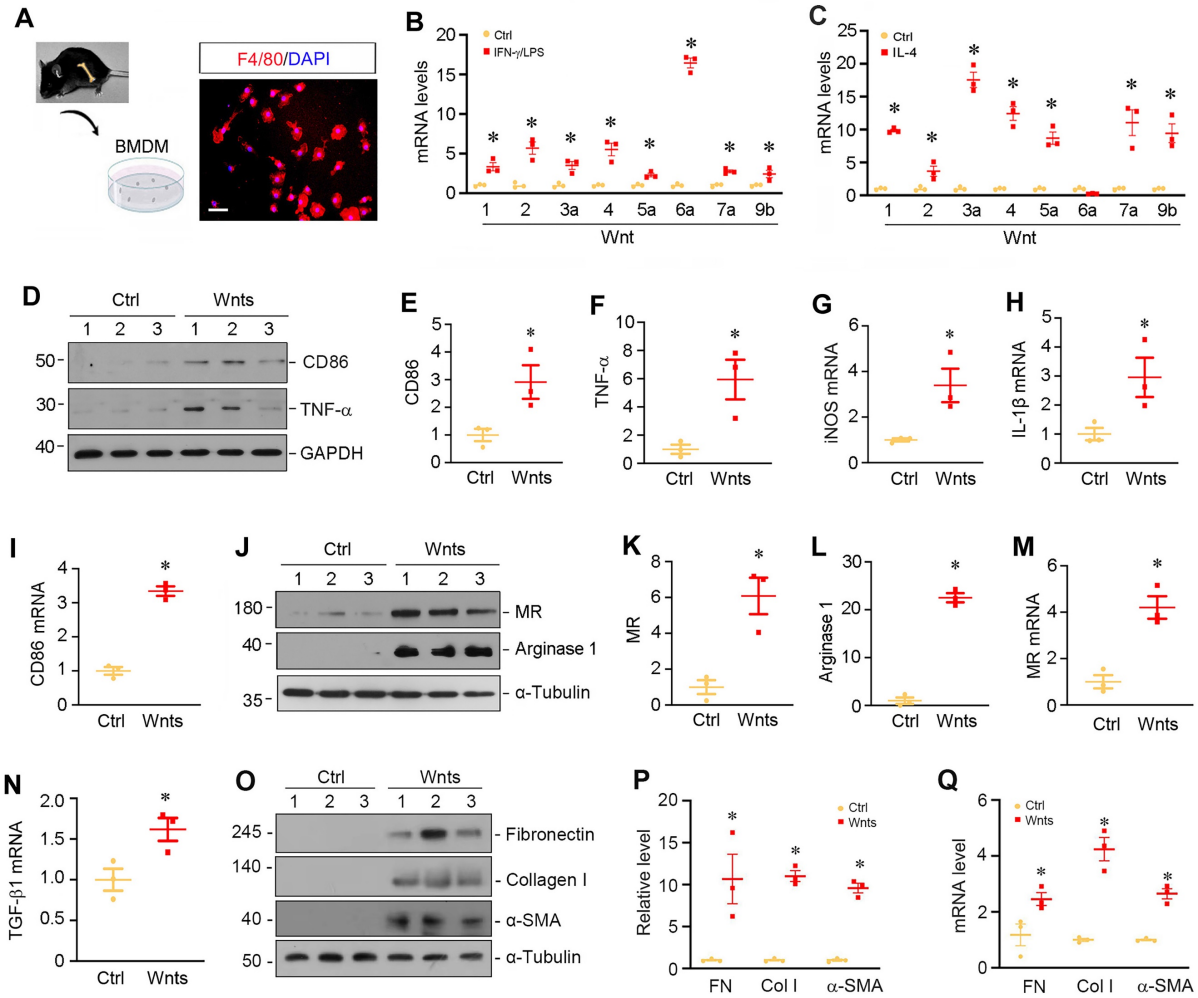
** Wnt upregulation is associated with macrophages polarization and activation *in vitro*.** (**A**) Characterization of the isolated bone marrow-derived macrophage (BMDM). Immunofluorescence staining for F4/80 showed the purity of cultured BMDMs. Scale bar, 25 µm. (**B**) BMDMs were induced to M1 polarization using of IFN-γ and LPS, and the expressions of various Wnts were detected by qRT-PCR. (**C**) BMDMs were induced to M2 polarization by IL-4, and the expressions of various Wnts were detected by qRT-PCR. (**D**) Western blot analyses showed that Wnts mixture induced M1 polarization by upregulating CD86 and TNF-α. (**E**-**F**) Quantitative data of CD86 and TNF-α in different groups as indicated. *P < 0.05 versus controls (n=3). (**G**-**I**) qRT-PCR showed that Wnts induced the mRNA expression of M1 polarization markers such as iNOS, IL-1β and CD86. (**J**) Western blotting showed that Wnts induced M2 polarization markers mannose receptor (MR) and arginase-1. (**K**, **L**) Quantitative data of MR and arginase 1 proteins. *P < 0.05 versus controls (n=3). (**M**, **N**) qRT-PCR showed that Wnts induced the mRNA expression of MR and TGF-β1. (**O**) Western blotting showed that Wnts induced fibrosis-related proteins such as fibronectin, collagen I and α-SMA in BMDMs. (**P**) Quantitative data of fibronectin (FN), collagen I (Col I) and α-smooth muscle actin (α-SMA) proteins. *P < 0.05 versus controls (n=3). (**Q**) qRT-PCR showed that Wnts induced the mRNA expression of FN, Col I and α-SMA. *P < 0.05 versus controls (n=3).

To validate the role of Wnts in macrophage activation, we isolated and cultured primary macrophages from bone marrow (BM) by using a defined differentiation protocol. As shown in Figure 2A, the purity of BM-derived macrophages (BMDMs) was confirmed by immunofluorescence staining for F4/80 protein. We then assessed the expressions of Wnts mRNA in different polarized macrophages induced by interferon-γ and lipopolysaccharides (IFN-γ/LPS) or IL-4, respectively. As shown in Figure 2B, multiple Wnt ligands such as Wnt1, Wnt2, Wnt3a, Wnt4, Wnt5a, Wnt6a, Wnt7a and Wnt9b were induced after BMDMs were polarized to M1 phenotype triggered by IFN-γ/LPS. Similarly, when BMDMs were polarized to M2 phenotype by IL-4, the mRNA levels of those Wnt ligands were upregulated as well (Figure 2C), whereas the mRNA of Wnt2b, Wnt7b, Wnt8a, Wnt8b, Wnt9a and Wnt16 was undetectable in the BMDMs after either IFN-γ/LPS or IL-4 treatment. As shown in Figure 2B and C, Wnt6a was mostly upregulated in M1 macrophages, while Wnt3a was highly induced by M2 subtype. To further assess the role of Wnts in macrophage polarization, we treated quiescent macrophages with a mixture of recombinant Wnt proteins (Wnt1 and Wnt3a). As shown in Figure 2D-F, Wnt proteins induced M1 polarization as shown by an upregulation of CD86 and TNF-α proteins. Similarly, Wnts also induced the mRNA expression of inducible nitric oxide synthase (iNOS), IL-1β and CD86 by qRT-PCR. However, Wnts appeared to have a stronger role in mediating M2 than M1 polarization and induced substantially high levels of MR and arginase 1 proteins in BMDMs (Figure 2, J-M). Furthermore, Wnts also induced the expression of TGF-β1 mRNA and the proteins and mRNA of several fibrosis-related proteins such as fibronectin, collagen I and α-smooth muscle actin (α-SMA) (Figure 2, N-Q), consistent with the notion of macrophage-myofibroblast transition (MMT) *in vitro* 11, 26.

### Myeloid-specific ablation of Wls inhibits macrophage activation

**Figure 3:**
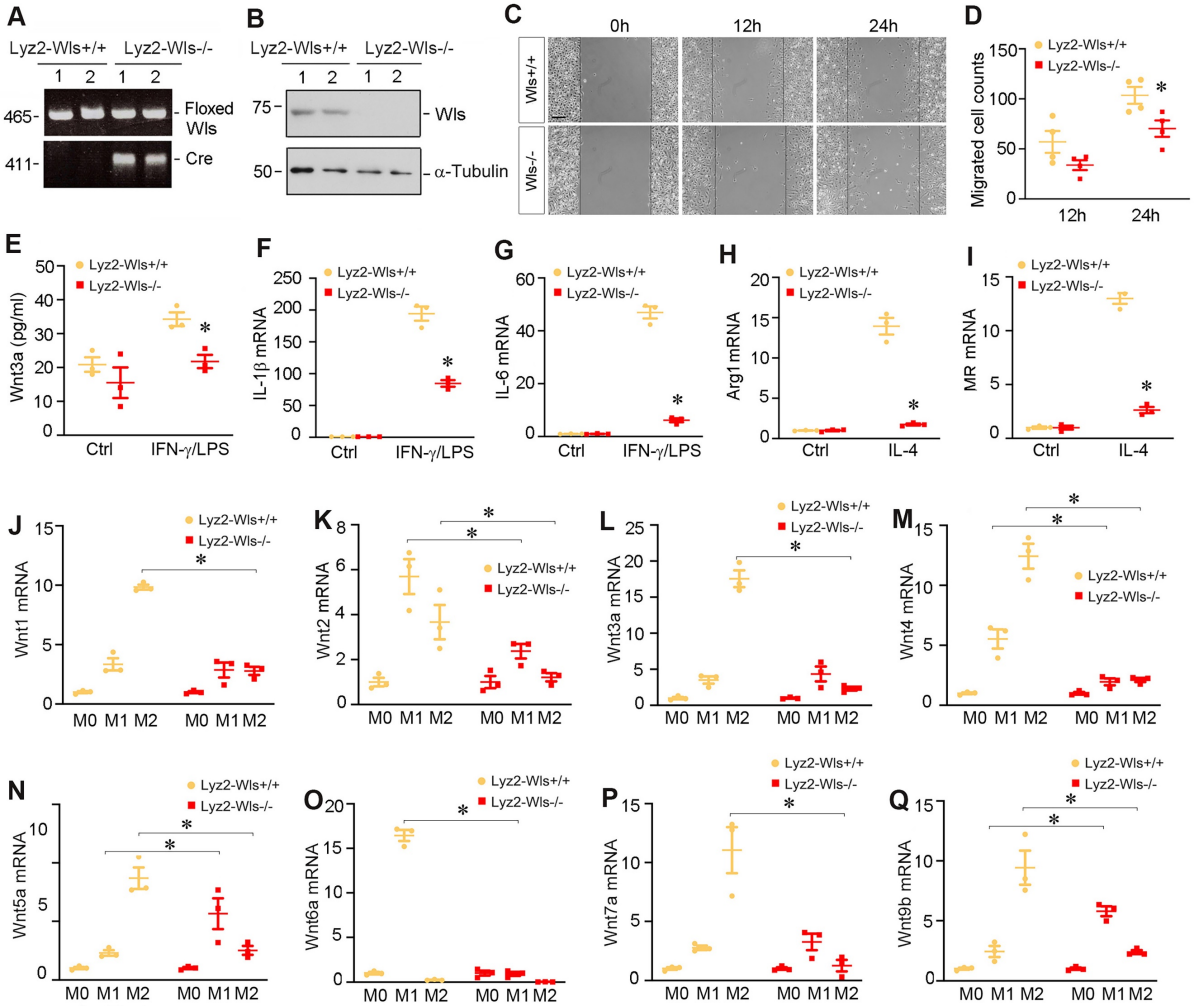
**Mice with myeloid-specific ablation of Wls inhibits macrophage polarization and activation.** (**A**) Mice genotyping was analyzed by PCR. Control Wls^fl/fl^ mice (Lyz2-Wls+/+) and myeloid-specific Wls knockout mice (Lyz2-Wls-/-) (genotype: Wls^fl/fl^, Cre^+^) are shown. (**B**) Western blot analysis demonstrated a substantial reduction of Wls protein in the BMDM of Lyz2-Wls-/- mice, comparing to Lyz2-Wls+/+ controls. (**C**, **D**) Wound-healing assay showed that loss of Wls reduced BMDM migration. Representive micrographs (**C**) and quantitation of migrated cells across the line (**D**) are shown. *P < 0.05 versus Lyz2-Wls+/+ controls (n = 4). Scale bar, 150 µm. (**E**) ELISA assay showed the levels of Wnt3a protein secreted by BMDMs in the absence or presence of IFN-γ and LPS. *P < 0.05 versus Lyz2-Wls+/+ controls (n = 3). (**F**, **G**) qRT-PCR demonstrated the mRNA levels of IL-1β and IL-6 in BMDMs from Lyz2-Wls+/+ and Lyz2-WLs-/- mice in the absence or presence of IFN-γ and LPS. *P < 0.05 versus Lyz2-Wls+/+ controls (n = 3). (**H**, **I**) qRT-PCR demonstrated the mRNA levels of arginase 1 (**H**) and MR (**I**) in BMDMs from the control and Lyz2-Wls-/- mice in the absence or presence of IL-4. *P < 0.05 versus Lyz2-Wls+/+ controls (n = 3). (**J**-**Q**) qRT-PCR demonstrated the mRNA levels of Wnt1 (**J**), Wnt2 (**K**), Wnt3a (**L**), Wnt4 (**M**), Wnt5a (**N**), Wnt6a (**O**), Wnt7a (**P**) and Wnt9b (**Q**) in BMDMs from the control and Lyz2-Wls-/- mice in the absence or presence of IFN-γ/LPS or IL-4, respectively. *P < 0.05 versus Lyz2-Wls+/+ controls (n = 3).

To study the role of Wnts in macrophage activation *in vivo*, we utilized conditional knockout mice in which *Wntless* (*Wls*) gene was selectively disrupted in myeloids via the Cre-LoxP system (Figure 3A). As shown in Figure 3B, BMDMs isolated from mice with myeloid-specific deletion of Wls (Lyz2-Wls-/-) lost Wls protein expression, compared to those from Lyz2-Wls+/+ mice. Of note, Lyz2-Wls-/- kidney displayed no overt abnormality under basal physiological conditions. As shown in Figure 3C and D, ablation of Wls in the BMDMs of Lyz2-Wls-/- mice attenuated their migration capacity, compared to Lyz2-Wls+/+ controls. We observed that M1 polarization induced by IFN-γ/LPS was associated with the secretion of Wnt3a into the supernatants of Wls+/+ BMDMs. However, loss of Wls in BMDMs reduced Wnt3a secretion (Figure 3E).

To further assess the role of Wnts secretion in macrophage activation, we examined the effect of Wls ablation on macrophage polarization after stimulation with IFN-γ/LPS or IL-4. As shown in Figure 3F and G, ablation of Wls largely blocked the induction of IL-1β and IL-6 mRNA in response to IFN-γ/LPS stimulation. Similarly, loss of Wls inhibited the mRNA expression of arginase 1 and MR in BMDMs in response to IL-4 stimulation (Figure 3H and I), suggesting that macrophage activation is dependent on Wnt signaling.

We found that blockade of Wnt secretion by ablating Wls also substantially inhibited the mRNA expression of various Wnt ligands in BMDMs during the induction of macrophage polarization (Figure 3J-Q). During M1 polarization induced by IFN-γ/LPS, induction of Wnt2, Wnt4 and Wnt6a mRNA in Wls-/- BMDMs was abolished, whereas loss of Wls appeared to induce Wnt5a and Wnt9b expression. However, when BMDMs were induced to undergo M2 polarization by IL-4, loss of Wls hampered the induction of all Wnts including Wnt1, Wnt2, Wnt3a, Wnt4, Wnt5a, Wnt7a and Wnt9b (Figure 3J-Q). These results suggest that Wnts induction at the mRNA level during M1 or M2 polarization is dependent on Wnt secretion in an autocrine fashion.

### Myeloid-specific ablation of Wls inhibits renal inflammation in UUO

**Figure 4:**
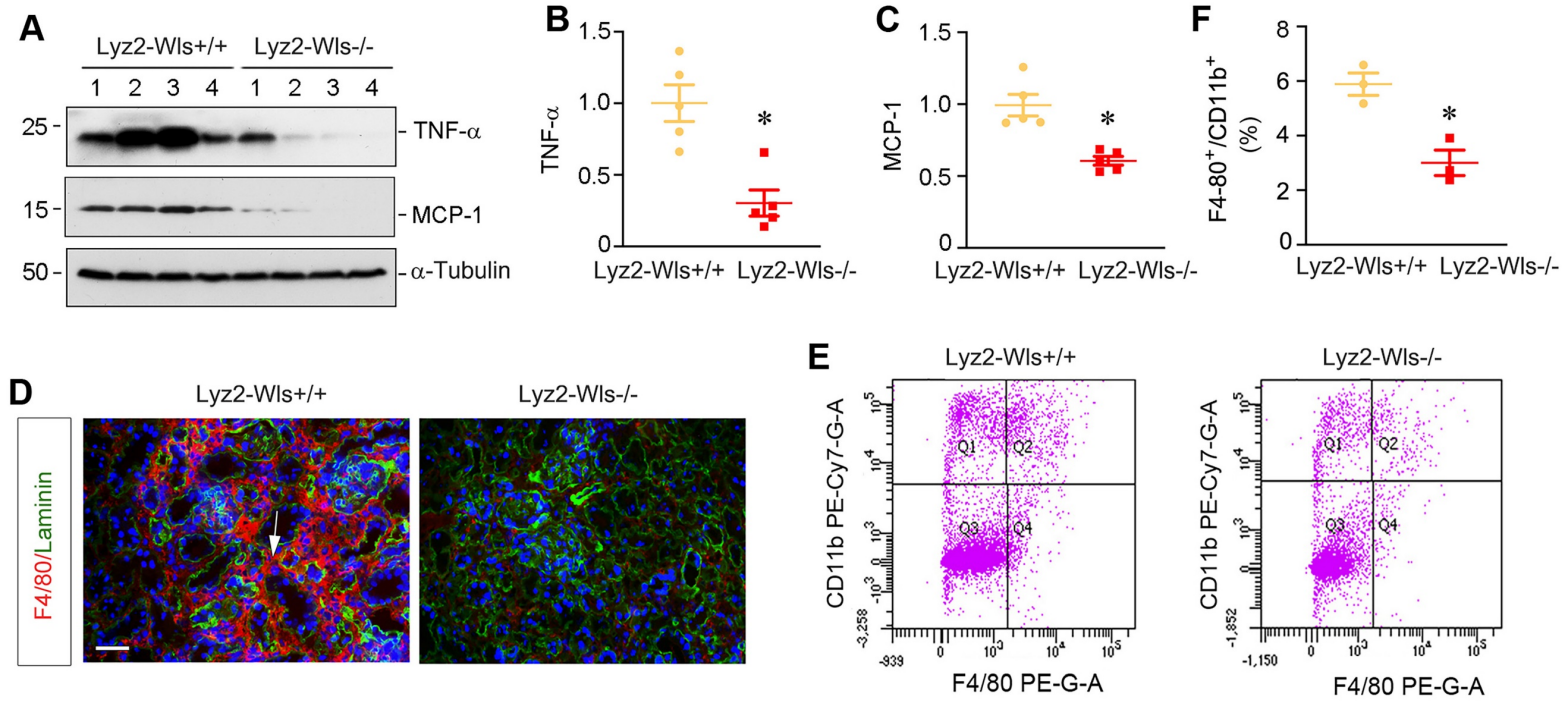
** Myeloid-specific deletion of Wls attenuatess kidney inflammation after obstructive injury.** (**A**-**C**) Western blot analyses demonstrate a decreased TNF-α and MCP-1 proteins in Lyz2-Wls-/- kidney after UUO, compared to Lyz2-Wls+/+ controls. Representative Western blot (**A**) and quantitative data (**B**, **C**) are presented. *P < 0.05 versus Lyz-Wls+/+ controls (n = 5). (**D**) Immunofluorescence staining for F4/80 in Lyz2-Wls+/+ and Lyz2-Wls-/- kidneys at 7 days after UUO. Sections were co-stained for laminin to outline tubular compartment. Arrow indicates positive staining. Scale bar, 50 µm. (**E**, **F**) Flow cytometry analysis of CD11b^+^F4/80^+^ macrophage from Lyz2-Wls+/+ and Lyz2-Wls-/- kidneys at 7 days after UUO. Quantitative data (**F**) are presented. *P < 0.05 versus Lyz2-Wls+/+ controls (n = 3).

To evaluate the role of macrophage-derived Wnts in renal inflammation *in vivo*, we utilized mouse model of UUO, in which renal infiltration of inflammatory cells is a major pathologic feature. To this end, Lyz2-Wls+/+ and Lyz2-Wls-/- mice were subjected to UUO and kidney tissues collected at 7 days after surgery. As shown in Figure 4A-C, renal expressions of TNF-α and monocyte chemoattractant protein-1 (MCP-1), also known as chemokine (CC-motif) ligand 2 (CCL2), was significantly downregulated in Lyz2-Wls-/- mice, compared to Lyz2-Wls+/+ controls. Furthermore, immunostaining showed that the infiltration of F4/80^+^ macrophages was also reduced in the obstructed kidney in Lyz2-Wls-/- mice, compared to Lyz2-Wls+/+ controls (Figure 4D). To quantitatively assess renal infiltration of macrophages, we used flow cytometry to measure F4/80^+^/CD11b^+^ macrophages in the obstructed kidney after UUO in Lyz2-Wls+/+ and Lyz2-Wls-/- mice. As shown in Figure 4E and F, significant less F4/80^+^/CD11b^+^ macrophages were found in the obstructed kidneys of Lyz2-Wls-/- mice, compared to Lyz2-Wls+/+ controls. These results suggest that loss of Wls in macrophages inhibits renal inflammation after obstructive injury.

### Myeloid-specific ablation of Wls inhibits β-catenin activation and kidney fibrosis in UUO

**Figure 5:**
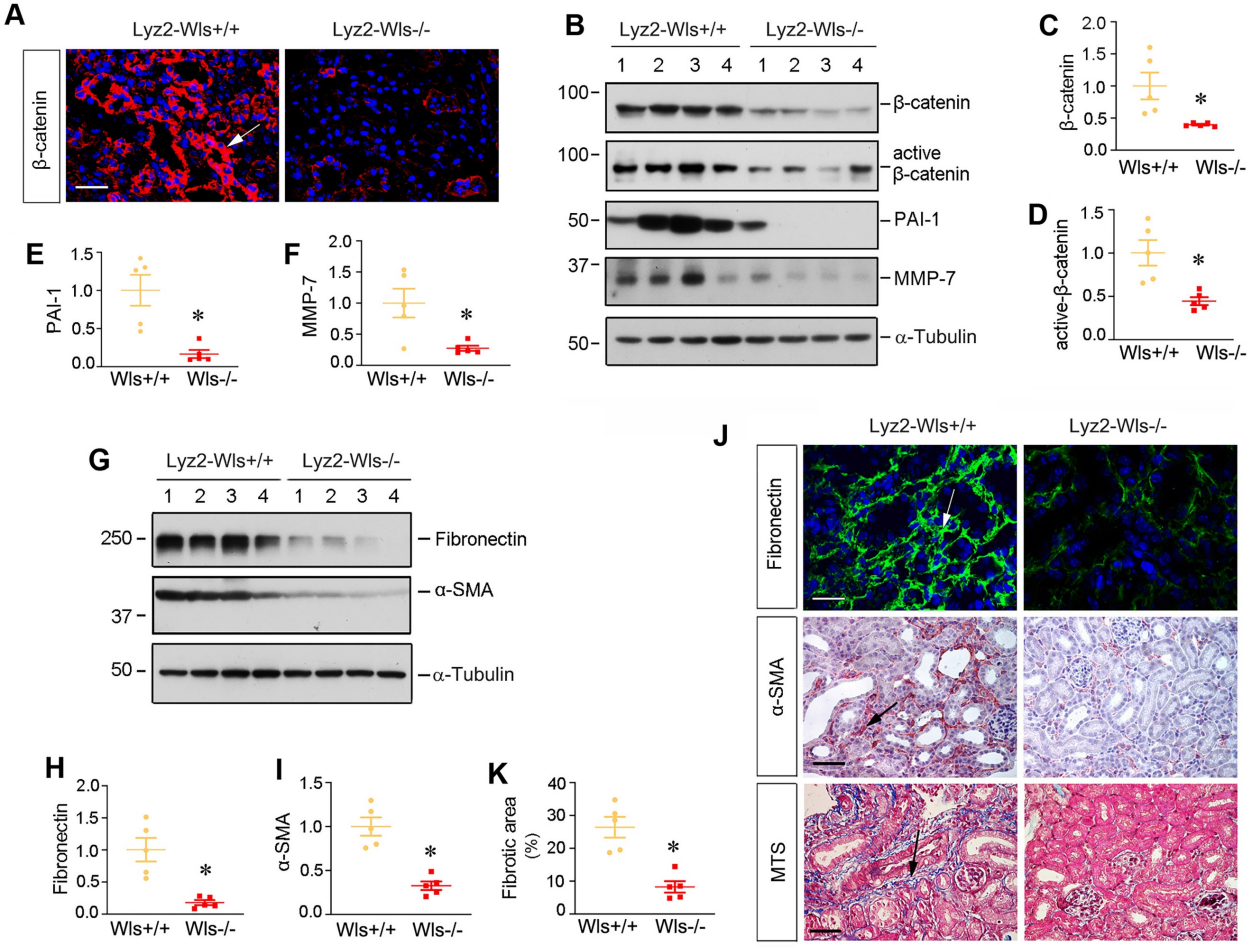
**Myeloid-specific ablation of Wls inhibits renal β-catenin activation and kidney fibrosis after obstructive injury.** (**A**) Immunofluorescence staining showed β-catenin expression and localization in the kidneys of Lyz2-Wls+/+ and Lyz2-Wls-/- mice at 7 days after UUO. Arrow indicates positive staining. Scale bar, 50 µm. (**B**) Western blot analyses of renal expression of β-catenin, active-β-catenin, PAI-1and MMP-7 proteins in the obstructive kidneys of Lyz2-Wls+/+ and Lyz2-Wls-/- mice at 7 days after UUO. (**C**-**F**) Quantitative data on the relative protein levels of β-catenin (**C**), active β-catenin (**D**), PAI-1 (**E**) and MMP-7 (**F**) in the obstructed kidney after UUO. *P < 0.05 versus Lyz2-Wls+/+ controls (n = 5). (**G**-**I**) Western blot analyses demonstrate a decreased fibronectin and α-SMA proteins in Lyz2-Wls-/- kidney after UUO, compared to Lyz2-Wls+/+ controls. Representative Western blot (**G**) and quantitative data (**H**, **I**) are presented. *P < 0.05 versus Lyz2-Wls+/+ controls (n = 5). (**J**) Representative micrographs show the expression and distribution of fibronectin and α-SMA after immunostaining and Masson's trichrome staining (MTS) for collagens in Lyz2-Wls+/+ and Lyz2-Wls-/- mice at 7 days after UUO. Arrows indicate positive staining. Scale bar, 50 µm. (**K**) Graphic presentation of quantitative data of fibrotic area in Lyz2-Wls+/+ and Lyz2-Wls-/- mice at 7 days after UUO. *P < 0.05 versus Lyz2-Wls+/+ controls (n = 5).

We then examined the effect of myeloid-specific ablation of Wls on kidney injury and fibrosis after UUO. To this end, we firstly assessed β-catenin expression in Lyz2-Wls-/- and Lyz2-Wls+/+ kidney after obstructive injury. As shown in Figure 5A-D, myeloid-specific ablation of Wls significantly inhibited the expression of total β-catenin and its active form. Immunostaining revealed that β-catenin in renal tubular epithelium was inhibited in Lyz2-Wls-/- kidney, compared to Lyz2-Wls+/+ controls, suggesting that macrophage-derived Wnts play an indispensable role in triggering β-catenin activation in renal tubules apparently via a paracrine fashion. Consistently, Western blot analysis showed that plasminogen activator inhibitor-1 (PAI-1) and matrix metalloproteinase-7 (MMP-7), the two downstream targets of β-catenin which are implicated in kidney fibrosis, were inhibited in Lyz2-Wls-/- kidney after UUO, compared to Lyz2-Wls+/+ controls (Figure 5B, E and F).

To directly link macrophage-derived Wnts to kidney fibrosis, we examined renal fibronectin and α- α-SMA expression by Western blotting and immunostaining. As shown in Figure 5G-I, renal expression of fibronectin and α-SMA was inhibited in Lyz2-Wls-/- kidney, compared to Lyz2-Wls+/+ controls. Consistently, immunostaining showed the same results as Western blotting (Figure 5J). Masson's trichrome staining (MTS) also showed a decreased collagen deposition in Lyz2-Wls-/- kidney (Figure 5K). These data indicate that macrophage-derived Wnts plays a pivotal role in the development and progression of renal fibrosis.

### Myeloid-specific ablation of Wls inhibits β-catenin signaling and kidney fibrosis after ischemic injury

**Figure 6:**
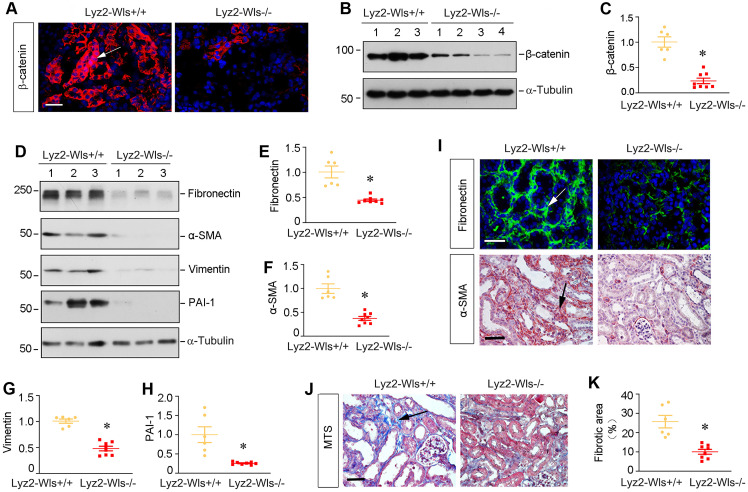
**Myeloid-specific ablation of Wls inhibits β-catenin activation and kidney fibrosis after ischemic injury.** (**A**) Representative micrographs show β-catenin expression and distribution in Lyz2-Wls+/+ and Lyz2-Wls-/- kidneys after UIRI. Arrow indicates positive staining. Scale bar, 50 µm. (**B**, **C**) Western blot analyses of renal expression of β-catenin in the kidneys of Lyz2-Wls+/+ and Lyz2-Wls-/- mice at 11 days after UIRI. Representative Western blot (**B**) and Quantitative data (**C**) are presented. *P < 0.05 versus Lyz-Wls+/+ controls (n = 6-8). (**D**-**H**) Western blot analyses demonstrate a decreased fibronectin, α-SMA, vimentin and PAI-1 proteins in Lyz2-Wls-/- kidney after UIRI, compared to Lyz2-Wls+/+ controls. Representative Western blot (**D**) and Quantitative data (**E**-**H**) are presented. *P < 0.05 versus Lyz2-Wls+/+ controls (n = 6-8). (**I**) Representative micrographs show immunostaining for fibronectin and α-SMA in the Lyz2-Wls+/+ and Lyz2-Wls-/- kidney at 11 days after UIRI. Arrow indicates positive staining. Scale bar, 50 µm. (J) Representative micrographs of Masson's trichrome staining (MTS) for collagens deposition in Lyz2-Wls+/+ and Lyz2-Wls-/- kidney at 11 days after UIRI. Arrow indicates positive staining. Scale bar, 50 µm. (**K**) Graphic presentation of quantitative data of fibrotic area in Lyz2-Wls+/+ and Lyz2-Wls-/- mice. *P < 0.05 versus Lyz2-Wls+/+ controls (n = 6-8).

To generalize the finding on the role of macrophage-derived Wnts in renal fibrosis, we employed another model of kidney fibrosis induced by unilateral ischemia-reperfusion injury (UIRI). Both Lyz2-Wls-/- and Lyz2-Wls+/+ mice were subjected to UIRI. Kidney tissues were collected at 11 days after surgery. As shown in Figure 6A-C, immunofluorescence staining and Western blot analyses showed that blockade of Wnts secretion from macrophages markedly reduced β-catenin abundance in the kidney after UIRI in Lyz2-Wls-/- mice, compared to Lyz2-Wls+/+ controls. Consistently, renal expression of fibronectin, α-SMA, vimentin and PAI-1 were also significantly downregulated in Lyz2-Wls-/- mice (Figure 6D-H). Immunostaining for fibronectin and α-SMA gave rise to the same results (Figure 6I). Masson's trichrome staining (MTS) also revealed that collagens deposition was ameliorated in the kidney of Lyz2-Wls-/- mice after UIRI, compared to Lyz2-Wls+/+ controls (Figure 6J and K). These data further confirm the crucial role of macrophage-derived Wnt in renal fibrosis.

### Macrophage-derived Wnts plays a central role in fibroblasts activation *in vitro*

**Figure 7:**
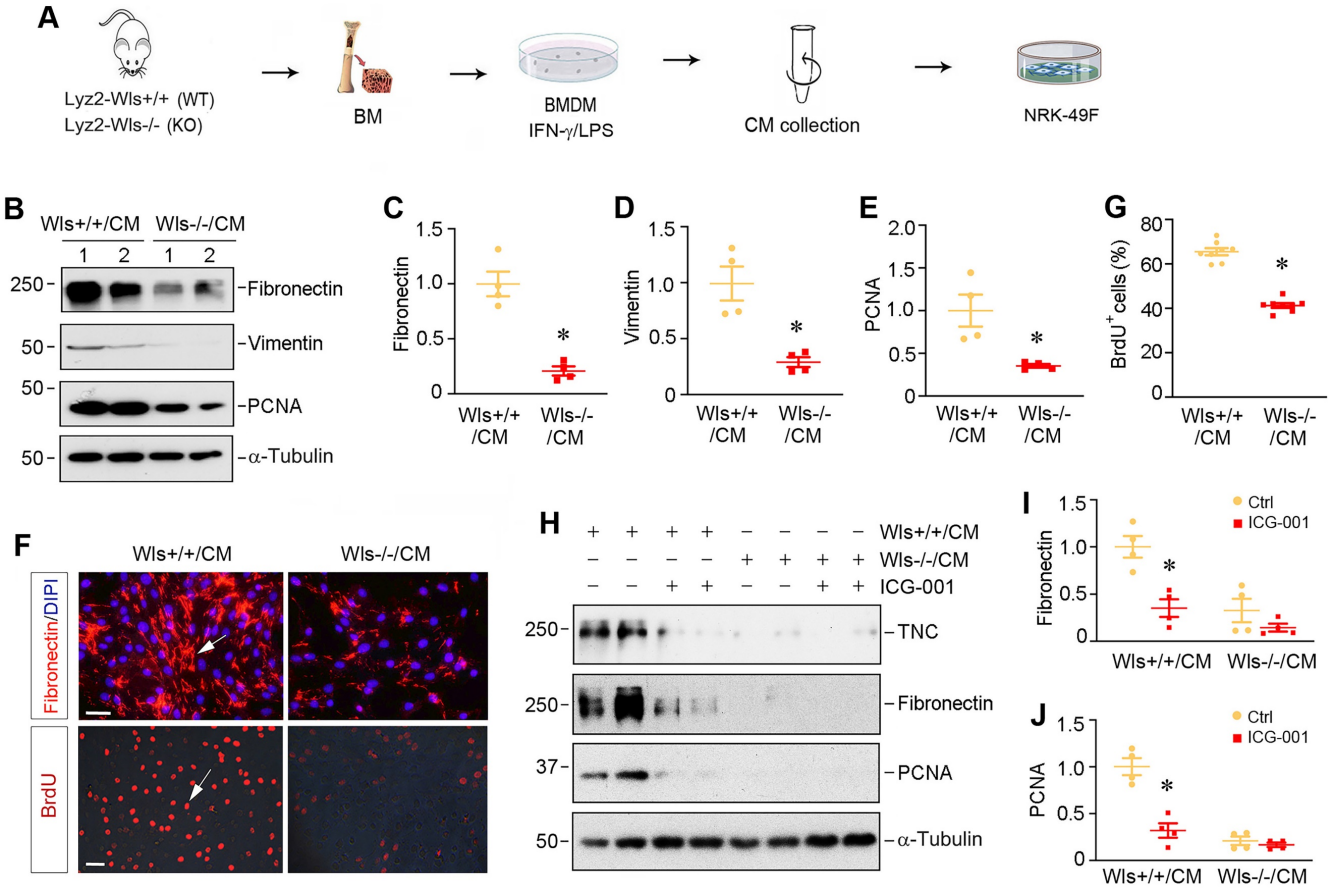
** Macrophage-derived Wnts plays a key role in fibroblasts activation.** (**A**) Experimental design. BMDM cells were collected from the Lyz2-Wls+/+ and Lyz2-Wls-/- mice. After BMDM cells were polarized by IFN-γ/LPS for 48 h, conditioned medium (CM) were harvested and used to stimulate NRK-49F fibroblast cells. (**B**-**E**) Western blot analyses show that conditioned medium derived from Wls-/- BMDM (Wls-/-/CM) inhibited the expression of fibronectin, vimentin and PCNA in NRK-49F cells, compared to the controls (Wls+/+/CM). Representative Western blot (**B**) and quantitative data (**C**-**E**) are presented. *P < 0.05 versus Wls+/+/CM controls (n = 3-4). (**F**) Immunostaining for fibronectin and BrdU in NRK-49F cells treated with Wls+/+/CM and Wls-/-/CM, respectively. Arrows indicate positive staining. Scale bar, 30 µm. (**G**) Quantitative data of BrdU staining are presented in panel (G). *P < 0.05 versus Wls+/+/CM controls (n = 8). (**H**-**J**) Western blot analyses show that inhibition of β-cateninn signaling by ICG-001 abolished fibroblast activation induced by Wls+/+/CM. NRK-49F cells were incubated with Wls+/+/CM or Wls-/-/CM in the absence or presence of ICG-001 for 48 h. Representative Western blot (**H**) and quantitative data (**I**, **J**) are presented. *P < 0.05 versus Wls+/+/CM controls (n = 4).

To explore how myeloid-specific Wls regulates kidney fibrosis, we investigated the effect of macrophages lacking Wls on the activation of renal interstitial fibroblasts in an *in vitro* system. As depicted in Figure 7A, BMDMs were isolated from Lyz2-Wls-/- and Lyz2-Wls+/+ mice, and induced toward M1 polarization by IFN-γ/LPS. The conditioned media (CM) were collected and used to treat the cultured normal rat kidney interstitial fibroblast cells (NRK-49F). As shown in Figure 7B-E, BMDM-CM from Lyz2-Wls-/- mice (Wls-/-/CM) significantly inhibited the expressions of fibronectin, vimentin and proliferating cell nuclear antigen (PCNA) in NRK-49F cells, compared to Wls+/+/CM controls. Consistently, immunofluorescence staining for fibronectin produced the same results (Figure 7F). The BrdU incorporation assay also confirmed that Wls-/-/CM hampered fibroblast DNA synthesis and proliferation, compared to Wls+/+/CM controls (Figure 7F and G).

To corroborate the role of macrophage-derived Wnts in mediating fibroblast activation, we then examined the effects of ICG-001, a small molecule that specifically inhibits β-catenin signaling. As shown in Figure 7H-J, ICG-001 abolished the induction of tenancin-C (TNC), fibronectin and PCNA expression by Wls+/+/CM, suggesting that macrophage-derived Wnts play a critical role in mediating fibroblasts activation and proliferation in the fibrotic kidney.

## Discussion

Macrophages are a subset of mononuclear phagocytic cells which originate from bone marrow to be tissue-resident, and they play pivotal role in tissue homeostasis and immune surveillance. Upon stimulation by tissue injury, macrophages are activated and polarized, and they then secret a host of proinflammatory and profibrotic cytokines and produce ECM components and other injurious molecules 12, 13. As such, they are not the bystander but one of the important players in the pathogenesis of human diseases including CKD 27, 28. In this study, we demonstrate that macrophage activation and polarization are associated with the simultaneous induction of multiple Wnt ligands. Furthermore, Wnt induction and subsequent secretion are obligatory for macrophage activation and polarization. We further show that conditional blockade of Wnt secretion in a myeloid-specific fashion in Lyz2-Wls-/- mice reduces macrophage activation and infiltration, inhibits myofibroblast activation and matrix production, and mitigates renal fibrotic lesions. These studies provide unambiguous evidence for a critical role of macrophage-derived Wnts in renal inflammation and fibrosis after kidney injury. Our findings for the first time illustrate that macrophages are one of the major sources of Wnt ligands in the injured kidney that promote renal fibrosis and CKD progression.

Macrophages are heterogenous cell populations with a high degree of plasticity and diversity 11. They are often classified into two major functional subsets, the classically activated (M1) or alternatively activated (M2) macrophages. M1-type macrophages play a strong role in the initiation and maintenance of inflammation since they secrete inflammatory cytokines including TNF-α, IL-1β, IL-6, and IFNγ 11, 28. Especially in the early stage of kidney injury, M1-type macrophages activation is predominant and destructive. The proinflammatory responses would induce tubular cell damage and cause the loss of renal function. The degree of M1 macrophages infiltration is associated with the severity of kidney injuries and serves as a key factor determining CKD progression 13, 29. In contrast to M1, M2-type macrophages are primarily involved in would healing and tissue remodeling. As such, macrophages exert their diverse roles in the initiation, progression and resolution of kidney fibrosis 14, 30. In this study, we show that Wnt/β-catenin signaling plays a pivotal role in stimulating both M1 and M2 macrophage activation and polarization (Figure 2), suggesting that this signaling is a master regulator that controls all processes of macrophage activation and differentiation in the injured kidney. By creating the conditional knockout mice in which Wls is deleted in a myeloid-specific fashion, we further demonstrate that macrophage-derived Wnts are obligatory for regulating the activation, differentiation and migration of macrophages (Figure 3 and 4). These findings are in line with a previous report that Wnt5a and Wnt6 are involved in macrophage differentiation and proliferation in mycobacterium tuberculosis infection 31. Of note, while blockade of Wnts secretion by deleting Wls in BMDMs inhibits most Wnts expression during macrophage activation, it actually induces Wnt5a and Wnt9b expression when BMDMs were induced toward M1 polarization (Figure 3N and Q). The exact reason behind this discrepancy remains unknown, but it suggests that Wnts are differentially regulated during macrophage M1 polarization.

Wnt/β-catenin is a developmental signaling pathway which is largely silent in adult kidneys but reactivated in various nephropathies 20, 32, 33. Notably, the activation of Wnt/β-catenin is often associated with accumulation of inflammatory cells such as macrophages. However, whether the Wnts produced by activated macrophages are required for the development and progression of kidney fibrosis remained to be elucidated. In this regard, the present study provides convincing evidence that macrophage-derived Wnts plays a pivotal role in the evolution of kidney fibrosis after either obstructive or ischemic injury (Figure 5 and Figure 6). *In vitro* co-culture studies also corroborate the important role of macrophage-derived Wnts in promoting interstitial fibroblasts proliferation and activation (Figure 7). These results underscore that myeloid-derived Wnts plays a central role in kidney fibrosis through promoting both macrophage polarization and fibroblasts activation and matrix production 34. Of interest, we found that Wnts also induce the expression of TGF-β1 and several key fibrosis-related proteins such as α-SMA, collagen I and fibronectin in BMDMs (Figure 2), suggesting that myeloid-derived Wnts may promote kidney fibrosis by activating macrophage-to-myofibroblast transition (MMT), a phenotypic conversion that plays an important role in renal fibrogenesis 11, 26. Although not tested, we speculate that blockade of Wnt secretion by macrophages may also alleviate tubular injury in CKD, as β-catenin expression in renal tubular epithelium was markedly downregulated in Lyz2-Wls-/- mice after injury as well (Figure 5 and 6). It has been reported that tubular β-catenin activation is known to induce cellular senescence 35, 36, which releases the senescence-associated secretory phenotype (SASP) leading to fibroblast activation. As such, a decreased β-catenin activation in renal tubular epithelium in the Lyz2-Wls-/- mice would prevent kidney from tubular cell senescence, thereby leading to renal protection.

It should be pointed out that a previous study reported the beneficial effects of Wnt7b induction in macrophages after AKI, which is associated with its ability to promote tubular cell proliferation and regeneration 17, 20. At first glance, it appears contradictory to our present study showing macrophage-derived Wnts contribute to renal inflammation and fibrosis after obstructive and ischemic injury (Figure 5 and 6). However, it is well known that Wnt/β-catenin excerts opposite actions in AKI versus in CKD. While β-catenin activation is protective by promoting injury repair and regeneration in AKI, sustained activation of this signaling leads to fibroblast activation and proliferation, thereby aggravating scar formation 20, 37. In this context, it is not surprising that macrophage-derived Wnts play an important role in macrophage polarization and interstitial fibroblast activation in diseased kidneys. It is also worthwhile to stress that, because the present study used Lyz2-Cre mice, Wnt secretion is blocked not only in macrophages but also in the neutrophils *in vivo*. Therefore, we cannot exclude the possibility that some renal protection in Lyz2-Wls-/- mice may be mediated by blocking Wnt secretion from neutrophils *in vivo*, although blockade of Wnt secretion from BMDMs is sufficient for suppressing macrophage and fibroblast activation *in vitro*.

In summary, we have shown herein that macrophage-derived Wnts are critically involved in mediating macrophage activation and polarization after kidney injury. Furthermore, blockade of Wnt secretion from macrophages represses the activation and expansion of interstitial fibroblasts and ameliorates renal fibrotic lesions. Although more studies are needed, our findings establish a crucial role of macrophage-derived Wnts in regulating macrophage polarization and fibroblast activation. These studies also offer significant insights into the intimate connection between renal inflammation and fibrosis after kidney injury.

## Materials and Methods

### Animal models

Homozygous *Wntless* floxed mice (C57BL/6J background) were obtained from the Jackson Laboratories (Stock #012888; Bar Harbor, ME). Transgenic mice that express Cre recombinase under the control of myeloid-specific lysozyme 2 gene (Lyz2) promoter (Lyz2-Cre) were also obtained from the Jackson (Stock #018956). By mating Wls floxed mice with Lyz2-Cre transgenic mice, conditional knockout mice (Lyz2-Wls-/-) in which *Wls* gene was specifically disrupted in myeloid cells (genotype: Wls^fl/fl^, Lyz2-Cre^+/-^) were created. These mice were cross-bred with homozygous Wls floxed mice (genotype: Wls^fl/fl^) to generate offspring with 50% myeloid-specific Wls knockout mice (Lyz2-Wls-/-) and 50% control Wls floxed mice (Lyz2-Wls+/+) within the same litters. A routine PCR protocol was used for genotyping of tail DNA samples. The PCR primer pairs used as follows: Cre transgene, 5'-AGGTGTAGAGAAGGCACTTAGC-3', which generated a 411 bp fragment; and *Wntless* genotyping, 5'-AGGCTTCGAACGTAACTGACC-3' and 5'-CTCAGAACTCCCTTCTTGAAGC-3', which yielded 556 bp band for the floxed alleles.

All animals were born normally at the expected Mendelian frequency, and they were normal in size and did not display any gross physical or behavioral abnormalities. Male mice at age of 8 weeks, approximately 22-25g in weight, were then subjected to UUO and UIRI by using established procedures, as described elsewhere 38, 39. Mice were sacrificed at day 7 after UUO. For UIRI model, mice were sacrificed at 11 days after surgery. Animal experiments were approved by the Animal Ethics Committee at the NanfangHospital, Southern Medical University.

### Quantitative, real-time RT-PCR

Total RNA was extracted using TRIzol RNA isolation system (Invitrogen). First strand cDNA synthesis was carried out by using a reverse transcription system kit according to the instructions of the manufacturer (Promega, Madison, WI). Quantitative, real-time RT-PCR (qRT-PCR) was performed on an ABI PRISM 7000 sequence detection system (Applied Biosystems, Foster City, CA) as described previously 38. The mRNA levels of various genes were calculated after normalizing with β-actin.

### Western blot analysis

Kidney tissues were lysed with radioimmunoprecipitation assay (RIPA) buffer containing 1% NP-40, 0.1% SDS, 100 μg/ml PMSF, 1% protease inhibitor cocktail, and 1% phosphatase I and II inhibitor cocktail (Sigma) in PBS on ice. The supernatants were collected after centrifugation at 13,000×*g* at 4°C for 15 min. Protein expression was analyzed by Western blot analysis, as previously described 40. The primary antibodies used were as follows: anti-Wls (MABS87, EMD Millipore, Billerica, MA), anti-Wnt1 (ab15251), anti-Wnt3a (SAB2108434 , Sigma), anti-active β-catenin (05-665, Millipore), anti-β-catenin (#610154; BD Transduction Laboratories, San Jose, CA), anti-MMP-7 (GTX104658, GeneTex), anti-PAI-1 (sc-5297), anti-mannose receptor (Abcam), anti-arginase 1 (Abcam), anti-CD86 (R&D system), anti-TNF-α (ab1793), anti-MCP-1 (PA5-34505, Thermo Scientific), anti-Vimentin (#2586; Cell Signaling Technology), anti-PCNA (sc-56) (Santa Cruz Biotechnology, Santa Cruz, CA), anti-fibronectin (F3648), anti-α-SMA (A2547), anti-collagen I (BA0325; Boster, Wuhan, China), anti-α-tubulin (T9026) (Sigma, St. Louis, MO), and anti-actin (MAB1501; EMD Millipore, Billerica, MA).

### Histology and immunohistochemical staining

Paraffin-embedded mouse kidney sections (3 µm thickness) were prepared by a routine procedure. The sections were subjected to Masson's trichrome staining (MTS) by standard protocol. Immunohistochemical staining was performed according to the established protocol as described previously 41. The primary antibodies against α-SMA (ab5694; Abcam, Cambridge, MA) and F4/80 (BIO-RAD) were used in the immunohistochemical staining.

### Immunofluorescence staining

Kidney cryosections were fixed with 3.7% paraformaldehyde for 15 min at room temperature and immersed in 0.2% Triton X-100 for 10 min. After blocking with 10% donkey serum in PBS for 1 h, slides were immunostained with following antibodies: β-catenin (ab15180), anti-fibronectin (F3648, Sigma, St. Louis, MO), anti-F4/80 (BIO-RAD), anti-laminin (L-8271, Sigma), vimentin (#5741s), anti-mannose receptor (Abcam). To visualize the primary antibodies, slides were stained with cyanine Cy2- or Cy3-conjugated secondary antibodies (Jackson ImmunoResearch Laboratories, West Grove, PA). Stained slides were viewed under a Leica TCS-SL confocal microscope equipped with a digital camera (Buffalo Grove, IL).

### Bone marrow derived macrophage (BMDM) preparation and polarization

Bone marrow cells were isolated from the tibia and femur of C57BL/6 mice. For their differentiation into BMDM, the extracted cells were incubated for 7 days in Petri dishes with 10% L929 conditioned media (source of M-CSF), 10% FBS and 1% penicillin/streptomycin (Sigma) in Dulbecco's Modified Eagle Media (DMEM). After 7 days, BMDMs were seeded into six-well cell culture plates. For M1 phenotype induction, BMDMs were treated with LPS (5 ng/ml) (Sigma) and IFN-γ (20 ng/ml) (PeproTech, China) for 24 h. For M2 phenotype induction, BMDMs were treated with IL-4 (20 ng/ml) (PeproTech, China) for 24 h.

### Cell culture and treatment

Normal rat kidney interstitial fibroblast (NRK-49F) cells were obtained from the American Type Culture Collection (Manassas, VA). Serum-starved NRK-49F cells were treated with conditioned medium of BMDM from Lyz2-Wls+/+ or Lyz2-Wls-/- mice in the absence or presence of ICG-001 (10 µM) for 48 h. After incubation for 48 h, NRK-49F cells were then collected and subjected to various analyses. Some cells were plated on sterile coverslips for immunofluorescence staining.

### BrdU incorporation assay

BrdU incorporation assay was performed according to our previous protocols 42. Briefly, cells were seeded onto 24-well plates and treated as indicated, and then pulsed with BrdU (10 mM) for 24 h. At the end of incubation, cells were fixed after removing the labeling medium, and cellular DNA was then denatured. Nonspecific binding was blocked by incubating the cells with 10% donkey serum for 30 min at room temperature. Incorporated BrdU was detected with mouse monoclonal anti-BrdU antibody (B2531; Sigma-Aldrich, St. Louis, MO), followed by incubation with cyanine Cy3-conjugated, affinity-purified secondary antibody (Jackson Immuno Research Laboratories, West Grove, PA). Stained samples were viewed under an Eclipse E600 epifluorescence microscope equipped with a digital camera (Nikon, Melville, NY).

### Isolation of kidney cells and flow cytometry

Kidneys were harvested, cut into small pieces, and incubated with collagenase type IV (Sigma) in RPMI-1640. After incubation and washing with PBS, cells were centrifuged at 4^o^C, 1800 rpm for 7 min. The pellet was gently suspended in 1640 medium, and passed through a 70-nm strainer (BD Falcon). About 5 ml Lympholyte-M was added to cell suspension and then centrifuged at 1200 g for 20 min. The lymphocytes were then collected, washed with PBS, and seeded in a 96-well plate at 100 µl/well, and stained with anti-CD11b-cy7, anti-F4/80 PE in 96-well plate on ice for 30 min. After washing, cells were fixed with fixation and permeabilization solution for 10 min in dark. Cells were then centrifuged at 4^o^C, 1800 rpm for 4 min. Detection of the cell surface antigens by flow cytometry was performed on a LSR 561 (Becton Dickinson) or FACS Calibur instrument (Becton Dickinson) with analysis using Flow Jo (Tree Star Inc.) or Cell Quest (Becton Dickinson) software.

### Statistical analyses

All data examined were expressed as mean ± SEM. Statistical analysis of the data was carried out using SPSS 13.0 (SPSS Inc, Chicago, IL). Comparison between groups was made using one-way ANOVA followed by Student-Newman-Kuels test or Dunnett's T3 procedure. *P* < 0.05 was considered significant.

## Abbreviations

AKI: acute kidney injury
BMDMs: bone marrow-derived macrophages
CKD: chronic kidney disease
ECM: extracellular matrix
FGF2: fibroblast growth factor-2
IL-1: interleukin-1
iNOS: inducible nitric oxide synthase
LPS: lipopolysaccharide
Lyz2: lysozyme 2, also known as lysozyme M
TGF-β: transforming growth factor-β
TNF-α: tumor necrosis factor-α
UIRI: unilateral ischemia-reperfusion injury
UUO: unilateral ureteral obstruction
Wls: Wntless
